# Gender-specific associations between polymorphisms of the circadian gene *RORA* and cutaneous melanoma susceptibility

**DOI:** 10.1186/s12967-021-02725-5

**Published:** 2021-02-06

**Authors:** Clara Benna, Senthilkumar Rajendran, Giovanna Spiro, Chiara Menin, Luigi Dall’Olmo, Carlo Riccardo Rossi, Simone Mocellin

**Affiliations:** 1 Department of Surgery Oncology and Gastroenterology, University of Padova, Padova, Italy; 2 First Surgical Clinic, Azienda Ospedaliera Padova, Padova, Italy; 3 Surgical Oncology Unit, Veneto Institute of Oncology (IOV-IRCCS), Padova, Italy; 4 Immunology and Diagnostic Molecular Oncology Unit, Veneto Institute of Oncology (IOV - IRCCS), Padova, Italy

**Keywords:** Cutaneous melanoma, Circadian clock, RORA, Susceptibility, Prognosis, Single nucleotide polymorphisms, SNP, Steroid hormone, Nuclear receptor

## Abstract

**Background:**

Melanoma is the deadliest of skin cancers and has an increasing annual incidence worldwide. It is a multi-factorial disease most likely arising from both genetic predisposition and environmental exposure to ultraviolet light. Genetic variability of the components of the biological circadian clock is recognized to be a risk factor for different type of cancers. Moreover, two variants of a clock gene, *RORA*, have been associated with melanoma patient’s prognosis. Our aim is to test the hypothesis that specific single nucleotide polymorphisms (SNPs) of the circadian clock genes may significantly influence the predisposition to develop cutaneous melanoma or the outcome of melanoma patients.

**Methods:**

We genotyped 1239 subjects, 629 cases of melanoma and 610 healthy controls in 14 known SNPs of seven selected clock genes: *AANAT*, *CLOCK*, *NPAS2*, *PER1*, *PER2*, *RORA*, and *TIMELESS*. Genotyping was conducted by q-PCR. Multivariate logistic regression was employed for susceptibility of melanoma assessment, modeled additively. Subgroup analysis was performed by gender. For the female subgroup, a further discrimination was performed by age. For prognosis of melanoma assessment, multivariate Cox proportional hazard regression was employed. The Benjamini–Hochberg method was utilized as adjustment for multiple comparisons.

**Results:**

We identified two *RORA* SNPs statistically significant with respect to the association with melanoma susceptibility. Considering the putative role of RORA as a nuclear steroid hormone receptor, we conducted a subgroup analysis by gender. Interestingly, the *RORA* rs339972 C allele was associated with a decreased predisposition to develop melanoma only in the female subgroup (OR 0.67; 95% CI 0.51–0.88; P = 0.003) while *RORA* rs10519097 T allele was associated with a decreased predisposition to develop melanoma only in the male subgroup (OR 0.62; 95% CI 0.44–0.87; P = 0.005). Moreover, the *RORA* rs339972 C allele had a decreased susceptibility to develop melanoma only in females aged over 50 years old (OR 0.67; 95% CI 0.54–0.83; P = 0.0002). None of the studied SNPs were significantly associated with the prognosis.

**Conclusions:**

Overall, we cannot ascertain that circadian pathway genetic variation is involved in melanoma susceptibility or prognosis. Nevertheless, we identified an interesting relationship between melanoma susceptibility and *RORA* polymorphisms acting in sex-specific manner and which is worth further future investigation.

## Background

Melanoma, which arises from the uncontrolled proliferation of melanocytes, is the deadliest of skin cancers and has an annual incidence of 22 new cases per 100,000 inhabitants in the US. It is a multi-factorial disease most likely arising from both genetic predisposition and environmental exposure to ultraviolet light. Phenotypical characteristics such as fair skin type, dysplastic nevi and multiple common nevi contribute to increase melanoma risk. In general, the predisposition to melanoma follows a polygenic model where in addition to the high penetrance genes with familial aggregation, as *CDKN2A* mutations, many low/medium penetrance genes are recognized with a risk-modifying role in the general population [1]. Approximately 5–10% of cutaneous melanoma cases occurs in a familial setting [2]. Genome-wide association studies (GWAS) and improved DNA sequencing techniques as next generation sequencing (NGS) contributed to elucidate the genetic basis of melanoma susceptibility with the identification of a plethora of new genetic loci associated with melanoma. Although, several genes responsible for melanoma predisposition have been identified, our knowledge on this field is still poorly understood [3].

An increasing number of epidemiological studies associates chronodisruption with the risk of developing various type of cancers, including melanoma [4, 5], leading the International Agency for Research on Cancer (IARC) to classify Shifwork (with night shifts, which involves circadian disruption) as a potential carcinogenic for humans (Group 2A) [6]. Regarding the relationship between the circadian clock machinery and melanoma, the majority of researchers focused on the expression of the circadian clock genes in melanoma cell lines and in skin biopsies in human or mouse. These studies showed that clock genes are downregulated in melanoma as compared to normal adjacent tissue (or compared to nevi) [7, 8]; moreover, the reduced clock gene expression is associated with increased tumor aggressiveness and a worse prognosis [7, 9]. Only one study addressed the issue on genetic variability of the promoter region of the *NPAS2* clock gene and melanoma risk, and found a significant association with a polymorphic GGC repeat [10]. Another study examined the association of polymorphisms of steroid hormone receptors and melanoma prognosis, and found significant associations between cutaneous melanoma-specific survival and two SNPs on the clock gene *RORA*, whose putative protein encodes a ligand-activated transcription factor [11].

In our previous analysis on genetic predisposition to cutaneous melanoma, we studied the copy number variations (CNVs) of the transcription factor *E2F1* [12] and found that 1.6% of melanoma patients harbored more than two copies of *E2F1.* The difference with the healthy subjects group was statistically significant. In the present article, we focused our attention on the role of single nucleotide polymorphisms (SNPs) of the components of the circadian system on melanoma biology. In particular, we intended to test the hypothesis that specific SNPs of the circadian clock genes, such as *AANAT* (arylalkylamine N-acetyltransferase), *CLOCK* (clock circadian regulator), *NPAS2* (neuronal PAS domain protein 2), *PER1* (period circadian clock 1), *PER2* (period circadian clock 2), *RORA* (retinoic acid-related orphan receptor A), and *TIMELESS* (timeless circadian clock) could significantly influence the predisposition to develop cutaneous melanoma or the outcome of melanoma bearing patients. To this aim, we genotyped 629 cases of melanoma and 610 healthy controls in 14 known SNPs of the above reported clock genes.

## Material and methods

### Study design

We conducted a retrospective study to test the hypothesis that genetic variants, such as SNPs, of the circadian pathway might be associated with the susceptibility or prognosis of patients affected with melanoma. To this aim, we extracted the clinico-pathological data of patients treated at our institution (Veneto Institute of Oncology, Italy) between 2008 and 2015, using a prospectively maintained database linked to our institutional biobank (Clinica Chirurgica I—University Hospital of Padova, Italy). To be included in the study, each case had to meet the following requirements: (1) histologically confirmed diagnosis of cutaneous melanoma or metastasis from melanoma; (2) pathology-based information on TNM stage; (3) follow-up data (minimum follow up: six months); (4) availability of peripheral blood for genotyping purposes.

### Patients and healthy donors

We retrospectively selected 629 consecutive melanoma patients, and 610 healthy controls. The selection of the latter was both population-based (n = 270 blood donors) and hospital-based (n = 340, healthy subjects who visited the Clinica Chirurgica I practice for routine check-ups). All patients signed an informed consent form explaining the research purposes of the blood withdrawal. Healthy controls dataset was already employed in our previous analyses [13, 14].

### SNPs selection

We focused on 5 core clock genes, which are *CLOCK*, *NPAS2*, *PER1*, *PER2*, and *RORA*. Moreover, we added two clock-related genes *TIMELESS*, associated with cancer risk in several studies [15–18] and *AANAT* for its role in the biosynthesis of melatonin, a key marker of the circadian system.

We selected either Tag SNPs, interrogating The Genome Variation Server of the University of Washington (http://gvs.gs.washington.edu/GVS/) and the TagSNP tool of the US National Institute of Environmental Health Sciences (https://snpinfo.niehs.nih.gov/snpinfo/snptag.html), or variants already known to be associated with cancer susceptibility or prognosis, that had a minor allele frequency > 5%. We relied on our previous meta-analysis [19], on our previous case–control studies [13, 14, 20] and on literature. The publications referring to cancer studies for each SNP are listed in Table 2.

DNA extraction and genotyping as described in [13].

### Statistical analysis

For susceptibility of melanoma assessment, multivariate logistic regression was employed, modeled additively by minor allele count. Odds ratios (OR) and 95% confidence intervals were used as a measure of association of each SNP. In those multivariate models, the evaluated outcome was the presence or absence of melanoma, while the explanatory variables were the single SNPs adjusted for age and gender.

Subgroup analysis was performed by gender. For the female subgroup, a further discrimination was performed by age (> 50 years old vs < 50 years old).

Sensitivity analysis was conducted excluding patients with melanoma of unknown primary (MUP).

For prognosis of melanoma assessment, multivariate Cox proportional hazard regression was employed. Overall survival was defined as the time from the date of tumor diagnosis to the date of death by any cause or last follow-up visit. Hazard ratios (HR) and 95% confidence intervals were used as a measure of association. In those multivariate models the evaluated event was the patient’s survival, the time to event were the months of survival, and the explanatory variables were the single SNP adjusted for age, gender and melanoma stage.

The Benjamini–Hochberg method (1995) was employed as adjustment for multiple comparisons (False Discovery Rate Online Calculator, 2016, Carbocation Corporation, https://tools.carbocation.com/FDR). False discovery rate (FDR) cut-off was set at 0.1.

Hardy-Weimberg Equilibrium (HWE) was tested for both samples (patients and healthy controls) for each SNP employing OEGE—Online Encyclopedia for Genetic Epidemiology studies [26], http://www.oege.org/software/hwe-mr-calc.shtml. This tool is a HWE calculator for biallelic SNPs based on Chi—square statistic.

Statistical power was calculated for each SNP employing the on-line tool “Power and Sample Size” of the University of Vanderbilt (http://biostat.mc.vanderbilt.edu/wiki/Main/PowerSampleSize) [27]. Power was defined as the probability of correctly rejecting the null hypothesis that the relative risk (OR) was equal to 1, given 629 case patients and 610 controls. The type I error probability α was set to 0.05 and ψ (OR considered clinically relevant in our study) was set to 0.80. Finally, the expression quantitative trait locus (eQTL) analysis was carried out interrogating the GTEx Portal (https://gtexportal.org/home/).

Rcmdr: R Commander. R package version 2.4–2 was employed for the analyses.

## Results

The analysis was based on a total of 1239 subjects, 629 cases of melanoma and 610 healthy controls, all of European ancestry. Thirty-four out of 629 patients had melanoma of unknown primary. The healthy control dataset was already employed in our previous analyses [13, 14], detailed subjects’ characteristics and the main features of the SNPs we investigated are reported in Tables 1 and 2, respectively.

**Table 1:** Clinico-pathological characteristics of 629 melanoma patients and 610 healthy donors

*Characteristic*	*Controls (n* = *610)*	*Cases (n* = *629)*
Mean age, years (st.dev.)	48.6 (14.8)	56.5 (15.7)
Gender, n (%)		
Male	336 (55.2)	329 (52.3)
Female	273 (44.8)	300 (47.7)
Female age, n (%)		
> 50 years	150 (54.9)	172 (57.3)
< 50 years	123 (45.1)	128 (42.7)
Source of Controls, n (%)		
Hospital	340 (55.7)	
Population	270 (44.3)	
Patient status, n (%)		
Alive		417 (66.3)
Deceased		212 (33.7)
Median survival, months (min, max)		73.3 (1.0, 340.6)
Primary tumor*,* n (%)		
Known		595 (94.6)
Unknown		34 (5.4)
Tumoral stage, n (%)		
I		174 (27.7)
II		159 (25.3)
III		267 (42.4)
IV		29 (4.6)

**Table 2:** Genotypes and main features of the circadian gene SNPs

Gene	SNP ID	Genotype	Controls N (%)	Cases N (%)	Chr	Region	Residue change	References in cancer
***AANAT***	rs3760138	TT	146 (24.3)	173 (27.7)	17	intron 5′-UTR		[28]
		TG	323 (53.7)	302 (48.3)				
		GG	132 (22.0)	150 (24.0)				
	rs11077821	CC	419 (69.1)	448 (71.5)	17	intron		[29]
		CT	175 (28.9)	166 (26.5)				
		TT	12 ( 2.0)	13 ( 2.1)				
***CLOCK***	rs1801260	TT	323 (53.2)	336 (53.4)	4	3′-UTR		[13, 14, 20, 22, 24, 30–34]
		TC	228 (37.6)	251 (39.9)				
		CC	56 ( 9.2)	42 ( 6.7)				
	rs3736544	GG	241 (39.8)	253 (40.2)	4	exon	Asn > Asn	
		GA	269 (44.5)	294 (46.7)				
		AA	95 (15.7)	82 (13.0)				
	rs3749474	CC	259 (42.6)	258 (41.0)	4	3′-UTR		[14, 19, 20, 28, 34]
		CT	266 (43.8)	280 (44.5)				
		TT	83 (13.7)	91 (14.5)				
***NPAS2***	rs2305160	GG	283 (46.7)	273 (44.0)	2	exon	Thr > Ala	[14, 16, 18, 23, 24, 28, 29, 34–43]
		GA	264 (43.6)	290 (46.7)				
		AA	59 ( 9.7)	58 ( 9.3)				
***PER1***	rs3027178	TT	281 (46.1)	262 (42.4)	17	exon	Thr > Thr	[14, 19, 25, 44]
		TG	253 (41.5)	279 (45.1)				
		GG	76 (12.5)	77 (12.5)				
***PER2***	rs934945	CC	386 (63.4)	410 (66.1)	2	exon	Gly > Glu	[13, 14, 19, 21–25, 44]
		CT	206 (33.8)	194 (31.3)				
		TT	17 ( 2.8)	16 ( 2.6)				
***PER2***	rs2304674	AA	330 (54.5)	349 (55.8)	2	intron		[23, 24, 29]
		AG	246 (40.7)	232 (37.1)				
		GG	29 ( 4.8)	45 ( 7.2)				
***RORA***	rs339972	TT	312 (51.4)	349 (55.7)	15	intron		[13, 14, 19, 24]
		TC	233 (38.4)	244 (38.9)				
		CC	62 (10.2)	34 ( 5.4)				
	rs10519097	CC	422 (69.3)	464 (74.4)	15	intron		[13, 14, 19, 24]
		CT	173 (28.4)	151 (24.2)				
		TT	14 ( 2.3)	9 ( 1.4)				
***TIMELESS***	rs3809125	CC	250 (41.4)	258 (41.0)	12	exon	Ile > Val	
		CT	280 (46.4)	297 (47.2)				
		TT	74 (12.3)	74 (11.8)				
	rs7302060	TT	181 (29.9)	192 (30.6)	12	upstream		[13–17, 19]
		TC	304 (50.2)	299 (47.6)				
		CC	121 (20.0)	137 (21.8)				
	rs774027	AA	157 (25.9)	164 (26.1)	12	intron		[13, 18, 19, 24, 39]
		AT	301 (49.7)	303 (48.2)				
		TT	148 (24.4)	162 (25.8)				

A total of 14 preselected SNPs in 7 circadian clock genes were successfully genotyped, and no departures from Hardy–Weinberg equilibrium were observed neither among the controls nor among the patients, as summarized in Additional file 1: Table S1. The mean statistical power for this analysis was approximately 70%; detailed statistical power for each SNP is reported in Additional file 1: Table S1.

## Susceptibility and prognosis assessment

Associations between the selected clock gene SNPs and melanoma susceptibility were tested assuming an additive model of inheritance. We used odds ratios (ORs) and their corresponding 95% confidence intervals (95% CI) to measure the strength of association between each polymorphism and melanoma susceptibility. The results are reported in Table 3.

**Table 3:** Associations of circadian pathway gene SNPs with susceptibility to melanoma under the additive model of inheritance

GENE	SNP ID	Minor allele	OR	95% CI	P-value
*AANAT*	rs3760138	G	0.98	[0.83—1.15]	0.770
	rs11077821	T	0.96	[0.76—1.20]	0.700
*CLOCK*	rs1801260	C	0.91	[0.76—1.09]	0.320
	rs3736544	A	0.97	[0.82—1.15]	0.717
	rs3749474	T	1.04	[0.88—1.23]	0.635
*NP2S2*	rs2305160	A	1.05	[0.88—1.25]	0.618
*PER1*	rs3027178	G	1.05	[0.89—1.25]	0.547
*PER2*	rs2304674	G	1.06	[0.87—1.28]	0.572
	rs934945	T	0.90	[0.72—1.11]	0.315
*RORA*	*rs339972*	*C*	*0.76*	*[0.63—0.91]*	*0.003*
	rs10519097	T	0.77	[0.61—0.97]	0.026
*TIMELESS*	rs3809125	T	0.99	[0.83—1.18]	0.907
	rs7302060	C	1.02	[0.87—1.20]	0.813
	rs774027	T	1.03	[0.87—1.21]	0.747

After adjusting for multiple testing, we identified two SNPs on *RORA* locus significantly associated with melanoma susceptibility. rs339972 C allele and rs10519097 T allele were associated with a decreased melanoma risk (OR 0.76; 95% CI 0.63–0.91; P = 0.003 and OR 0.77; 95% CI 0.61–0.97; P = 0.026, respectively). Adjusted p-values are reported in Additional file 1: Table S2.

No differences were found excluding from the analyses the 34 patients with melanoma of unknown primary.

Considering the putative role of RORA as a nuclear steroid hormone receptor, we decided to conduct a subgroup analysis by gender. The results are reported in Table 4.

**Table 4:** Associations of circadian pathway genes with susceptibility to melanoma under the additive model of inheritance. Stratification by gender and by age

**GENE**	**SNP ID**	**FEMALES**			**MALES**		
		**OR**	**95%CI**	**P-value**	**OR**	**95%CI**	**P-value**
*AANAT*	rs3760138	0.95	[0.75–1.21]	0.693	0.98	[0.77–1.24]	0.850
	rs11077821	0.89	[0.64–1.25]	0.505	1.03	[0.75–1.41]	0.874
*CLOCK*	rs1801260	0.90	[0.69–1.17]	0.423	0.96	[0.74–1.24]	0.724
	rs3736544	0.75	[0.59–0.96]	0.021	1.24	[0.98–1.57]	0.074
	rs3749474	1.33	[1.05–1.68]	0.018	0.79	[0.62–1.01]	0.058
*NPAS2*	rs2305160	1.09	[0.84–1.41]	0.529	0.97	[0.75–1.24]	0.789
*PER1*	rs3027178	0.94	[0.74–1.20]	0.633	1.11	[0.87–1.41]	0.416
*PER2*	rs2304674	1.12	[0.86–1.47]	0.405	1.01	[0.76–1.32]	0.971
	rs934945	0.87	[0.64–1.19]	0.393	0.93	[0.68–1.26]	0.618
*RORA*	rs339972	*0.67*	*[0.51–0.88]*	*0.003*	0.84	[0.65–1.09]	0.181
	rs10519097	0.93	[0.67–1.30]	0.673	*0.62*	*[0.44–0.87]*	*0.005*
*TIMELESS*	rs3809125	1.05	[0.82–1.34]	0.701	0.95	[0.74–1.21]	0.668
	rs7302060	1.00	[0.79–1.26]	0.994	1.07	[0.85–1.35]	0.564
	rs774027	1.05	[0.84–1.33]	0.659	0.97	[0.77–1.22]	0.805
*Age*							
	< 50 years						
* RORA*	rs339972	0.84	[0.68–1.04]	0.100			
	rs10519097	0.83	[0.63–1.09]	0.180			
	> 50 years						
*RORA*	*rs339972*	*0.67*	*[0.54–0.83]*	*0.0002*			
	rs10519097	0.83	[0.64–1.08]	0.158			

Interestingly, the *RORA* rs339972 C allele was associated with a decreased melanoma susceptibility only in the female subgroup (OR 0.67; 95% CI 0.51–0.88; P = 0.003) while *RORA* rs10519097 T allele was associated with a decreased predisposition to develop melanoma only in the male subgroup (OR 0.62; 95% CI 0.44–0.87; P = 0.005). A further subgroup analysis was carried out dividing the female dataset by age at diagnosis (> 50 years old vs < 50 years old) to discriminate if menopausal or premenopausal status could interact with *RORA* variants and melanoma associations. Females aged over 50 years old carrying *RORA* rs339972 C allele had a decreased susceptibility to develop melanoma of 33% (OR 0.67; 95% CI 0.54–0.83; P = 0.0002). No other tested associations were statistically significant after correcting for multiple testing.

No statistically significant associations with prognosis were observed as reported, for each SNP, in Additional file 1: Table S3.

### eQTL search

The expression quantitative trait locus (eQTL) analysis was carried out interrogating the GTEx Portal. A significant result (P = 0.000015) was found for *RORA* rs339972 in pancreas, while no significant eQTLs were identified for *RORA* SNP rs10519097.

## Discussion

### Summary

In the present study, we tested the hypothesis that DNA genetic variations of the circadian clock genes might influence the susceptibility to develop cutaneous melanoma or the outcome of melanoma patients. The hypothesis stemmed from the growing number of studies correlating the risk or the prognosis of different tumour types with the genetic variability of the circadian system [19]. Moreover, cutaneous melanoma and the circadian system share a common denominator: ultraviolet (UV) radiation. The persistent exposure to sunlight, resulting in sunburn, especially in childhood, is an acknowledged melanoma risk factor [45]. Of note, light is the principal *zeitgeber* (literally *time giver*) of the biological clock, and thus plays a key role in clock synchronization. We selected 14 single nuclear polymorphisms in seven clock-related genes and we genotyped 1239 subjects, 629 melanoma patients and 610 healthy donors. Our results indicated that two SNPs of the *RORA* clock gene are associated with melanoma susceptibility. In particular, subgroup analysis revealed that rs339972 was statistically significant with respect to the association with melanoma susceptibility in females, while rs10519097 in males. Moreover, females carrying *RORA* rs339972 C allele aged over 50 years had a decreased susceptibility to develop melanoma of 33%. No associations were found with melanoma prognosis.

### RORA structure

RORA (also known as NR1F1, nuclear receptor subfamily 1, group F, member 1) belongs to the nuclear steroid hormone receptor family of transcription factors. It was identified in the 1990s for its sequence similarity with the retinoid acid receptor (RAR) [46, 47]. Receptors without their ligands were called orphan-receptors. More recently, the crystal structure of its ligand binding domain identified cholesterol and cholesterol sulphate as putative ligands [48, 49]. The *RORA* locus maps to human chromosome 15q22.2 and spans 730 kb genomic region, comprised of 15 exons. Alternative splicing and alternative promoter usage generate four isoforms RORA1-4, differently expressed in a tissue-specific manner. Different isoforms of RORA have different binding specificities and strengths of transcriptional activity [47]. In the early 2000s RORA was shown to display a rhythmic pattern of expression in a circadian manner in the liver, kidney, retina, lung and suprachiasmatic nuclei, the region of the brain responsible for circadian rhythmicity control. Few years later, RORA was shown to be necessary for the rhythmic circadian behavior, indeed mice deficient in RORA display an aberrant circadian rhythmicity [50]. In the clock machinery, RORA competes with other nuclear receptors REV-ERBα and β (NR1D1 and 2, nuclear receptor subfamily 1, group D, member 1 and 2, respectively) to bind specific DNA response element (RORE) in the promoter of the core clock gene *BMAL1* (brain and muscle Arnt-like protein-1, also known as ARNTL1); RORA activates, while REV-ERBα and β suppress *BMAL1* transcription [51–53]. Moreover, melatonin, a major clock output, putatively regulates RORA [54].

Both *RORA* studied variants, rs10519097 and rs33997, are located within introns (first and second respectively) of *RORA* transcript 1, referred to as *RORA1*, while they are upstream to transcripts 2–4, *RORA2-4*.

### Circadian clock and melanoma

Approximately ten years ago, a circadian clock was found also within the skin [55], in particular the different cellular subtypes, fibroblasts, keratinocyte and melanocytes have a local circadian machinery which includes the expression of RORA [56]. In cutaneous melanoma it has been described a general reduction of clock genes abundance compared to normal adjacent tissues [8]. RORA was found to be downregulated in melanoma compared to nevi, and the expression was directly correlated to overall and disease-free survival [7]. Moreover, non-metastatic cutaneous melanoma induces chronodisruption in central and peripheral circadian clocks [9]. Recently, two variants in the *RORA* locus were found to be associated with melanoma prognosis, rs782917 and rs17204952 as well as rs7253062 in *DNMT1*, a steroid hormone receptor as well. Combined analysis of risk genotypes of the three variants revealed a decreased cutaneous melanoma specific survival in a dose–response manner. The authors performed a pathway-based analysis to evaluate genetic variants of 191 steroid hormone-related genes [11]. This research rationale was based on the shared idea that melanoma represent a steroid-hormone related malignancy [57].

### Sex hormones and melanoma

Beside the mentioned risk factors, additional variables are ethnicity, age and gender, in the latter a female advantage has been generally revealed. Lifestyle, X chromosome dosage and sex hormones play a role in this disparity [58]. It has been reported that incidence rates of cutaneous melanoma rise steeply in women until about age 50, suggesting estrogen as a possible risk factor. Moreover, a cumulative dose-dependent increased risk of cutaneous melanoma was shown with the use of estrogens, oral contraceptives and hormonal replacement therapy, [59] and the risk of premenopausal melanoma may be increased among women who are current oral contraceptive users, particularly among those with longer durations of use [60]. An epidemiological study reported that the incidence of pregnancy-associated melanoma increases with increasing maternal age (women aged 40–55 had a 7.55-fold higher risk than women aged 15–24) [61]. Women with a severe teenage acne history, reflecting a hormone imbalance, as a higher circulating level of free testosterone, had an increased relative risk of melanoma [62]. In males, personal history of prostate cancer, neoplasia in which androgens play a major role, was found to be associated with an increased risk of melanoma [63].

### Sexually dimorphic regulation of RORA

Male and female sex hormones differentially and reciprocally regulate RORA, with androgen suppressing RORA while estrogen enhancing RORA expression. Dihydrotestosterone (DHT) and estradiol increase the binding of androgen receptor (AR) and estrogen receptor (ER) respectively to the *RORA* promoter region, and RORA in turn increases testosterone levels. The resultant negative feedback transcriptionally regulates aromatase, which converts male hormones to estrogens [64].

### Possible RORA SNPs interactions with melanoma susceptibly in a gender-specific manner

The results of the present study are supporting the role of hormone receptors in melanoma susceptibility. In particular, the analyzed *RORA* polymorphisms have different effect on melanoma susceptibility whereas rs339972 minor allele has a protective effect on premenopausal women while rs10519097 minor allele has a protective effect exclusively in men. A speculation could be that those polymorphisms interact differently with sex hormones and RORA expression, given the general knowledge that clock genes as well as RORA are down regulated in cancer including melanoma. Another hypothesis might be that the effect of different genetic variations is related to different *RORA* isoforms or to different RORA functions; beside the involvement in circadian rhythmicity, RORs molecules play a critical role in development, immunity and cellular metabolism [53]. Further information on these genetic variants derives from our previous studies. In our previous meta-analysis [19], rs339972 was statistically significant with respect to the association with cancer in general while rs10519097 with breast cancer risk. In our previous case–control studies [13, 14, 20] we found rs339972 to be associated with both gastric cancer and sarcoma susceptibility. In all those studies, the minor allele C had a protective effect, but the associations were statistically less robust (in terms of P-value) as compared to the association revealed by the present work. Noticeably, in our previous meta-analysis subgroups meta-analysis employing two datasets (4587 subjects) showed that the association of rs10519097 with breast cancer was significant with an intermediate level of evidence (summary OR: 0.85, CI: 0.75–0.96, P = 0.008). The A allele was associated with a reduced risk of developing cancer also in postmenopausal breast cancer patients (3700 subjects; summary OR: 0.87, CI: 0.75–1, P = 0.04) [19]. Nonetheless, further studied will be needed to explain and understand the mechanisms underlying our results.

The GTEx Portal was queried for the expression quantitative trait locus (eQTL) analysis. A significant result (P = 0.000015) was found for *RORA* rs339972 in pancreas, nevertheless a GWAS [65] revealed no significant associations with pancreatic cancer risk (OR: 0.92, CI: 0.85–1.00, P = 0.04). Further analyses are necessary to unravel the biological and clinical significance of those results.

## Conclusions

To the best of our knowledge, this is one of the few pioneering analyses investigating the relationship between cutaneous melanoma susceptibility or prognosis and circadian gene variants. Nonetheless, we must acknowledge some limitations of our work. First, we tested a limited number of clock genes and for each of them evaluated only selected SNPs, based on our previous analyses or on literature, therefore studies involving a larger amount of genes and SNPs are eagerly awaited. Second, all the enrolled subjects were of Caucasian ethnicity. Additional studies including multiple ethnicity should be performed in order to validate our hypothesis also in people with different ancestry. Third, the source of controls was both population and hospital based (University Hospital of Padova, Italy), nevertheless we choose patients with different conditions for the hospital-based fraction (i.e. patients treated for haemorrhoids, goitre, gastritis) to avoid selection bias. Fourth, we employed only one genetic model (that is, the additive model), whereas neither the recessive nor the dominant model were explored: however, our aim was not to identify the best genetic model for specific polymorphisms but rather to summarize (in a quantitative fashion) the evidence regarding the selected genetic variants.

Overall, we cannot conclude in favour of the hypothesis that variability of the circadian clock genes may affect melanoma susceptibility or prognosis, but we did find an interesting relationship between melanoma biology and *RORA* gene variants, which we believe is worth further investigation.

## Supplementary Information

**Additional file 1: Table S1.** Hardy-Weinberg equilibrium (HWE) test and detailed statistical power for each SNP analysis. **Table S2.** Benjamini-Hochberg adjusted p-values for each SNP analysis. **Table S3.** Associations of circadian pathway genes with prognosis of 629 melanoma patients under the additive model of inheritance.

## Data Availability

All data generated or analysed during this study are included in this published article [and its additional information files].

## Abbreviations

SNP: Single nucleotide polymorphism
GWAS: Genome-wide association studies
NGS: Next generation sequencing
IARC: International Agency for Research on Cancer
OR: Odds ratios
HR: Hazard ratios: 95%CI: 95% confidence interval
MUP: Melanoma of unknown primary
eQTL: Expression quantitative trait locus
FDR: False discovery rate
HWE: Hardy-Weimberg Equilibrium
Chr: Chromosome
RAR: Retinoid acid receptor
DHT: Dihydrotestosterone
AR: Androgen receptor
ER: Estrogen receptor
