# Supplementary material for: Gender-specific associations between polymorphisms of the circadian gene RORA and cutaneous melanoma susceptibility
Source: J Transl Med. 2021 Feb 6;19:57. doi: 10.1186/s12967-021-02725-5 (PMC7866430; doi:10.1186/s12967-021-02725-5)
Supplement: Supplementary file 1 — Additional file 1: Table S1. Hardy-Weinberg equilibrium (HWE) test and detailed statistical power for each SNP analysis. Table S2. Benjamini-Hochberg adjusted p-values for each SNP analysis. Table S3. Associations of circadian pathway genes with prognosis of 629 melanoma patients under the additive model of inheritance. [file 12967_2021_2725_MOESM1_ESM.docx]

**Additional FILE**

**MANUSCRIPT: Gender-specific associations between polymorphisms of the circadian gene *RORA* and cutaneous melanoma susceptibility**

AUTHORS: Clara Benna, Senthilkumar Rajendran, Giovanna Spiro, Chiara Menin, Luigi Dall’Olmo, Carlo Riccardo Rossi, Simone Mocellin

**Supplementary Table S1** Hardy-Weinberg equilibrium (HWE) test and detailed statistical power for each SNP analysis

|  |  |  | **HWE** | | | | | | **POWER** |
| --- | --- | --- | --- | --- | --- | --- | --- | --- | --- |
|  |  |  | **Controls** | | | **Cases** | | |  |
| **Gene** | **SNP ID** | **Minor allele** | **MAF** | **chi square** | **P-val** | **MAF** | **chi square** | **P-val** |  |
| AANAT | rs3760138 | G | 0.49 | 3.42 | 0.06 | 0.48 | 0.65 | 0.42 | 0.79 |
| AANAT | rs11077821 | T | 0.16 | 1.65 | 0.20 | 0.15 | 0.27 | 0.60 | 0.51 |
| CLOCK | rs1801260 | C | 0.28 | 2.85 | 0.09 | 0.27 | 0.28 | 0.60 | 0.68 |
| CLOCK | rs3736544 | A | 0.38 | 1.88 | 0.17 | 0.36 | 0.06 | 0.81 | 0.76 |
| CLOCK | rs3749474 | T | 0.36 | 1.23 | 0.27 | 0.37 | 1.12 | 0.29 | 0.75 |
| NPAS2 | rs2305160 | A | 0.32 | 0.05 | 0.82 | 0.33 | 2.32 | 0.13 | 0.71 |
| PER1 | rs3027178 | G | 0.33 | 2.57 | 0.11 | 0.35 | 0.04 | 0.84 | 0.73 |
| PER2 | rs934945 | T | 0.20 | 2.90 | 0.09 | 0.18 | 1.53 | 0.22 | 0.57 |
| PER2 | rs2304674 | G | 0.25 | 3.94 | 0.05 | 0.26 | 0.57 | 0.45 | 0.65 |
| RORA | rs339972 | C | 0.29 | 3.46 | 0.06 | 0.25 | 1.06 | 0.30 | 0.69 |
| RORA | rs10519097 | T | 0.17 | 0.58 | 0.45 | 0.14 | 0.70 | 0.40 | 0.52 |
| Timeless | rs3809125 | T | 0.35 | 0.10 | 0.75 | 0.35 | 0.67 | 0.41 | 0.74 |
| Timeless | rs7302060 | C | 0.45 | 0.11 | 0.74 | 0.46 | 1.03 | 0.31 | 0.78 |
| Timeless | rs774027 | T | 0.49 | 0.02 | 0.89 | 0.50 | 0.84 | 0.36 | 0.79 |
|  |  |  |  |  |  |  |  |  |  |
|  |  |  |  |  |  |  |  | MEAN | 0.69 |

Hardy-Weimberg Equilibrium (HWE) was tested for both samples (patients and healthy controls) for each SNP employing OEGE - Online Encyclopedia for Genetic Epidemiology studies [27], http://www.oege.org/software/hwe-mr-calc.shtml. This tool is a HWE calculator for biallelic SNPs based on Chi - square statistic.

Statistical power was calculated for each SNP employing the on-line tool “Power and Sample Size” of the University of Vanderbilt (http://biost at.mc.vande rbilt .edu/wiki/Main/Power Sampl eSize) [28]. Power was defined as the probability of correctly rejecting the null hypothesis that the relative risk (OR) was equal to 1, given 629 case patients and 610 controls. The type I error probability α was set to 0.05 and ψ (OR considered clinically relevant in our study) was set to 0.80.

**Supplementary Table S2** Benjamini-Hochberg adjusted p-values for each SNP analysis

| **SNP ID** | **Subgroup** | **Rank** | **Original P value** | **Critical Value** | **Benjamini-Hochberg Adjusted P value** | **Significant using an FDR of 0.1?** |
| --- | --- | --- | --- | --- | --- | --- |
| **RORA-rs339972** | **age >50, gender =F** | **1** | **0.00019** | **0.00156** | **0.01184** | **Yes** |
| **RORA-rs339972** | **OVERALL** | **2** | **0.00274** | **0.00313** | **0.08768** | **Yes** |
| **RORA-rs339972** | **gender=F** | **3** | **0.00349** | **0.00469** | **0.07445** | **Yes** |
| **RORA-rs10519097** | **gender=M** | **4** | **0.00524** | **0.00625** | **0.08384** | **Yes** |
| CLOCK-rs3749474 | gender=F | 5 | 0.01760 | 0.00781 | 0.22528 | No |
| CLOCK-rs3736544 | gender=F | 6 | 0.02090 | 0.00938 | 0.22293 | No |
| RORA-rs10519097 | OVERALL | 7 | 0.02590 | 0.01094 | 0.23680 | No |
| CLOCK-rs3749474 | gender=M | 8 | 0.05770 | 0.01250 | 0.46160 | No |
| CLOCK-rs3736544 | gender=M | 9 | 0.07420 | 0.01406 | 0.52764 | No |
| RORA-rs339972 | age <50, gender =F | 10 | 0.10400 | 0.01563 | 0.66560 | No |
| RORA-rs10519097 | age >50, gender =F | 11 | 0.15800 | 0.01719 | 0.91927 | No |
| RORA-rs10519097 | age <50, gender =F | 12 | 0.18000 | 0.01875 | 0.96000 | No |
| RORA-rs339972 | gender=M | 13 | 0.18100 | 0.02031 | 0.89108 | No |
| NP2S2-rs2305160 | prognosis | 14 | 0.27310 | 0.02188 | 1.00000 | No |
| RORA-rs10519097 | prognosis | 15 | 0.27690 | 0.02344 | 1.00000 | No |
| PER1-rs3027178 | prognosis | 16 | 0.30150 | 0.02500 | 1.00000 | No |
| AANAT-rs3760138 | prognosis | 17 | 0.31480 | 0.02656 | 1.00000 | No |
| PER2-rs934945 | OVERALL | 18 | 0.31500 | 0.02813 | 1.00000 | No |
| CLOCK-rs1801260 | OVERALL | 19 | 0.32000 | 0.02969 | 1.00000 | No |
| AANAT-rs11077821 | prognosis | 20 | 0.34270 | 0.03125 | 1.00000 | No |
| TERT-rs2736098 | prognosis | 21 | 0.35740 | 0.03281 | 1.00000 | No |
| PER2-rs934945 | gender=F | 22 | 0.39300 | 0.03438 | 1.00000 | No |
| PER2-rs2304674 | gender=F | 23 | 0.40500 | 0.03594 | 1.00000 | No |
| CLOCK-rs3749474 | prognosis | 24 | 0.40720 | 0.03750 | 1.00000 | No |
| PER1-rs3027178 | gender=M | 25 | 0.41600 | 0.03906 | 1.00000 | No |
| CLOCK-rs1801260 | gender=F | 26 | 0.42300 | 0.04063 | 1.00000 | No |
| PER2-rs2304674 | prognosis | 27 | 0.43670 | 0.04219 | 1.00000 | No |
| AANAT-rs11077821 | gender=F | 28 | 0.50500 | 0.04375 | 1.00000 | No |
| Timeless-rs7302060 | prognosis | 29 | 0.51180 | 0.04531 | 1.00000 | No |
| RORA-rs339972 | prognosis | 30 | 0.52080 | 0.04688 | 1.00000 | No |
| NPAS2-rs2305160 | gender=F | 31 | 0.52900 | 0.04844 | 1.00000 | No |
| PER1-rs3027178 | OVERALL | 32 | 0.54700 | 0.05000 | 1.00000 | No |
| Timeless-rs774027 | prognosis | 33 | 0.55380 | 0.05156 | 1.00000 | No |
| Timeless-rs7302060 | gender=M | 34 | 0.56400 | 0.05313 | 1.00000 | No |
| PER2-rs2304674 | OVERALL | 35 | 0.57200 | 0.05469 | 1.00000 | No |
| CLOCK-rs3736544 | prognosis | 36 | 0.57630 | 0.05625 | 1.00000 | No |
| TERT-rs2242652 | prognosis | 37 | 0.60550 | 0.05781 | 1.00000 | No |
| NPAS2-rs2305160 | OVERALL | 38 | 0.61800 | 0.05938 | 1.00000 | No |
| PER2-rs934945 | gender=M | 39 | 0.61800 | 0.06094 | 1.00000 | No |
| PER1-rs3027178 | gender=F | 40 | 0.63300 | 0.06250 | 1.00000 | No |
| CLOCK-rs3749474 | OVERALL | 41 | 0.63500 | 0.06406 | 0.99122 | No |
| Timeless-rs774027 | gender=F | 42 | 0.65900 | 0.06563 | 1.00000 | No |
| Timeless-rs3809125 | gender=M | 43 | 0.66800 | 0.06719 | 0.99423 | No |
| RORA-rs10519097 | gender=F | 44 | 0.67300 | 0.06875 | 0.97891 | No |
| Timeless-rs3809125 | prognosis | 45 | 0.68610 | 0.07031 | 0.97579 | No |
| AANAT-rs3760138 | gender=F | 46 | 0.69300 | 0.07188 | 0.96417 | No |
| AANAT-rs11077821 | OVERALL | 47 | 0.70000 | 0.07344 | 0.95319 | No |
| Timeless-rs3809125 | gender=F | 48 | 0.70100 | 0.07500 | 0.93467 | No |
| CLOCK-rs3736544 | OVERALL | 49 | 0.71700 | 0.07656 | 0.93649 | No |
| CLOCK-rs1801260 | gender=M | 50 | 0.72400 | 0.07813 | 0.92672 | No |
| TERT-rs2736100 | prognosis | 51 | 0.72640 | 0.07969 | 0.91156 | No |
| PER2-rs934945 | prognosis | 52 | 0.74470 | 0.08125 | 0.91655 | No |
| Timeless-rs774027 | OVERALL | 53 | 0.74700 | 0.08281 | 0.90204 | No |
| AANAT-rs3760138 | OVERALL | 54 | 0.77000 | 0.08438 | 0.91259 | No |
| NPAS2-rs2305160 | gender=M | 55 | 0.78900 | 0.08594 | 0.91811 | No |
| Timeless-rs774027 | gender=M | 56 | 0.80500 | 0.08750 | 0.92000 | No |
| Timeless-rs7302060 | OVERALL | 57 | 0.81300 | 0.08906 | 0.91284 | No |
| CLOCK-rs1801260 | prognosis | 58 | 0.83530 | 0.09063 | 0.92171 | No |
| AANAT-rs3760138 | gender=M | 59 | 0.85000 | 0.09219 | 0.92203 | No |
| AANAT-rs11077821 | gender=M | 60 | 0.87400 | 0.09375 | 0.93227 | No |
| Timeless-rs3809125 | OVERALL | 61 | 0.90700 | 0.09531 | 0.95161 | No |
| TERT-rs2853676 | prognosis | 62 | 0.95650 | 0.09688 | 0.98735 | No |
| PER2-rs2304674 | gender=M | 63 | 0.97100 | 0.09844 | 0.98641 | No |
| Timeless-rs7302060 | gender=F | 64 | 0.99400 | 0.10000 | 0.99400 | No |

The Benjamini-Hochberg method (1995) was employed as adjustment for multiple comparisons (False Discovery Rate Online Calculator, 2016, Carbocation Corporation, https://tools.carbocation.com/FDR). False discovery rate (FDR) cut-off was set at 0.1.

**Supplementary Table S3** Associations of circadian pathway genes with prognosis of 629 melanoma patients under the additive model of inheritance

| **SNP ID** | **HR** | **95%CI** | **P-Value** |
| --- | --- | --- | --- |
| AANAT-rs3760138 | 1.17 | [0.86 - 1.60] | 0.31 |
| AANAT-rs11077821 | 1.16 | [0.86 - 1.56] | 0.34 |
| CLOCK-rs1801260 | 1.02 | [0.83 - 1.27] | 0.84 |
| CLOCK-rs3736544 | 1.06 | [0.87 - 1.28] | 0.58 |
| CLOCK-rs3749474 | 0.92 | [0.76 - 1.12] | 0.41 |
| NP2S2-rs2305160 | 0.89 | [0.71 - 1.10] | 0.27 |
| PER1-rs3027178 | 0.90 | 0.74 - 1.10] | 0.30 |
| PER2-rs2304674 | 0.90 | [0.68 - 1.18] | 0.44 |
| PER2-rs934945 | 0.96 | [0.75 - 1.23] | 0.74 |
| RORA-rs339972 | 0.93 | [0.74 - 1.17] | 0.52 |
| RORA-rs10519097 | 0.85 | [0.63 - 1.14] | 0.28 |
| Timeless-rs3809125 | 1.04 | [0.85 - 1.28] | 0.69 |
| Timeless-rs7302060 | 1.07 | [0.88 - 1.29] | 0.51 |
| Timeless-rs774027 | 0.94 | [0.78 - 1.14] | 0.55 |

For prognosis of melanoma assessment, multivariate Cox proportional hazard regression was employed. Overall survival was defined as the time from the date of tumor diagnosis to the date of death by any cause or last follow-up visit. Hazard ratios (HR) and 95% confidence intervals were used as a measure of association. In those multivariate models the evaluated event was the patient’s survival, the time to event were the months of survival, and the explanatory variables were the single SNP adjusted for age, gender and melanoma stage.
